# LRP5 knockdown: effect on prostate cancer invasion growth and skeletal metastasis *in vitro* and *in vivo*

**DOI:** 10.1002/cam4.111

**Published:** 2013-09-05

**Authors:** Shafaat A Rabbani, Ani Arakelian, Riaz Farookhi

**Affiliations:** 1 Department of Medicine, McGill University Health CentreMontreal, Quebec, Canada; 2 Department of Physiology, McGill UniversityMontreal, Quebec, Canada

**Keywords:** Bone metastasis, gene expression, prostate cancer, wnt signaling

## Abstract

Prostate cancer (PCa) is a common hormone-dependent malignancy associated with the development of skeletal metastases. This is due to the increased expression of a number of growth factors, cytokines, and proteases which collectively drive the metastatic cascade in general and increased propensity to develop skeletal metastasis in particular. While a number of signaling pathways have been implicated in PCa progression, the highly complex wnt/β-catenin pathway is unique due to its ability to regulate gene expression, cell invasion, migration, survival, proliferation, and differentiation to contribute in the initiation and progression of PCa. Members of the wnt family bind to the Frizzle proteins or lipoprotein-related receptor proteins 5, 6 (LRP5, -6) to activate this key pathway. In the current study, we have investigated the role of wnt/β-catenin pathway in PCa progression, skeletal metastasis, and gene expression using the dominant negative plasmid of LRP5 (DN-LRP5) and human PCa cells PC-3. Inactivation of LRP5 resulted in mesenchymal to epithelial shift, lack of translocation of β-catenin to cell surface, increased tumor cell proliferation, decreased colony formation, migration and invasion *in vitro*. These effects were attributed to decreased expression of pro-invasive and pro-metastatic genes. In *in vivo* studies, PC-3-DN-LRP5 cells developed significantly smaller tumors and a marked decrease in skeletal lesion area and number as determined by X-ray, micro (μ) CT and histological analysis. Collectively results from these studies demonstrate the dominant role of this key pathway in PCa growth and skeletal metastasis and its potential as a viable therapeutic target.

## Introduction

Prostate cancer (PCa) is a leading cause of cancer-associated death in men. In the multistep process of tumor progress, PCa starts as a low-invasive hormone-sensitive (androgen) cancer which over time becomes hormone insensitive and acquires an aggressive phenotype 1,2. These cellular characteristics of PCa cells have led to the use of antiandrogens as standard therapy for PCa patients 1,2. However, in late-stage PCa when the majority of tumor cells are hormone insensitive these therapies become ineffective. In this late stage, tumor cells invade through the extracellular matrix via hematogenous or lymphatic routes and seed at distant organs 3–6. PCa is unique among malignancies due to the high incidence of developing skeletal metastases 7,8. While these skeletal lesions are primarily osteolytic in breast cancer, they are osteoblastic or mixed in PCa patients 7,8. In order to fully understand the mechanism of developing skeletal metastases and bone remodeling in PCa, the role of a number of growth factors, proteases, and cytokines have been described which are regulated via multiple intracellular signaling pathways 3–6. Among these the Wnt/β-catenin signaling pathway which is well characterized for its role in health and disease state has been proposed as a significant player in PCa progression 9,10.

Members of the *wnt* family act as ligands for the β-catenin which regulates cell adhesion and signal transduction via its ability to act as a transcriptional activator which can form complexes with DNA-binding proteins 11,12. It was first identified for its role in colon cancer by forming complexes with the tumor suppressor adenomatous polyposis gene (APC) which was followed by characterization of β-catenin mutation in patients with colon cancer 13,14. Subsequently, a large number of studies provided compelling evidence by demonstrating the alerted expression of a number of genes of this pathway in several common malignancies 15–23. Members of the *wnt* family bind to frizzle protein or lipoprotein-related receptor proteins 5 and 6 (LRP5/-6) via the canonical pathway leading to the stabilization of cytosolic β-catenin which translocates to the nucleus to regulate the expression of a number of genes implicated in bone biology and tumor progression 24. LRP5 and -6 are unique among their family of receptor proteins due to the absence of an internalization sequence which can promote endocytosis 25. However, the extracellular part contains epidermal growth factor like sequence repeats which can promote interactions with other proteins and cell membrane 26. While mutations in these regions of LRP5 have been associated with change in bone mass, the ligand proteins for LRP5 in the bone remains to be identified 27–29. Mutation of LRP5 results in osteoporosis and increased bone mass syndrome, whereas conditional deletion of the *LRP5* gene in mice results in enhanced bone formation 30. In patients with PCa, at least 5% of tumors showed mutations of β-catenin 31–33. In previous studies we have shown increased expression of wnt-1 and β-catenin in invasive PCa cell lines and in primary PCa specimens; levels which were significantly higher in patients with skeletal metastases which collectively led to demonstrate the increased role of this pathway in hormone refractory PCa 34. Using multiple human PCa cell lines the expression of members of this pathway was demonstrated 34. Both LRP5 and -6 are implicated in mediating Wnt/β-catenin signaling; however, due to the overwhelming role of LRP5 in tumor progression and bone biology, we targeted this gene to define its role in PCa-associated skeletal metastasis 35,36.

In the current study, we have directly examined the role of β-catenin pathway by transfection of DN-LRP5 plasmid into a human PCa cells PC-3 which represent invasive PCa. While recent studies have shown that PC-3 cells may not best represent hormone refractory PCa cells, they have been extensively used to evaluate the role of several genes in tumor progression and skeletal metastasis which was the primary focus of our studies. The effect of transfection of DN-LRP5 plasmid and abrogation of β-catenin pathway was examined on PC-3 cells characteristics *in vitro* and on tumor growth and skeletal metastasis *in vivo*.

## Material and Methods

### Cell culture

Human PCa cell line, PC-3, was obtained from the American Type Culture Collection and maintained in RPMI 1640 with 10% fetal bovine serum, 2 mmol/L l-glutamine, and 100 units/mL penicillin/streptomycin sulfate. PC-3 cells were transfected with vector alone (PC-3-pcDNA3.1) or dominant negative plasmid encoding LRP5 (DN-LRP5), a kind gift from Dr. Mathew Warman, Boston, MA. Following transfection of control and experimental plasmids, cells were selected by maintaining them in culture in the presence of 500 μg/mL of G418. At least 5 monoclonal cell lines expressing control plasmid and DN-LRP5 were maintained in culture. These stably transfected cell lines were pooled to avoid any artifacts associated with transfection of plasmids and used for all further studies.

### Quantitative real-time PCR

Total cellular RNA from PC-3 cells and cells transfected with vector alone or DN-LRP5 plasmid was extracted using TRIzol (Invitrogen Life Technologies, Burlington, ON) according to the manufacturer's protocol. Two micrograms of total RNA was used for reverse transcription and amplification. Two microliter of cDNA was used in a 20 μL reaction with SYBR green master mix, 0.5 μmol/L forward and reverse primers. Reaction was performed in an ABI StepOnePlus (Applied Biosystems, Burlington, ON) Real Time PCR system with the following conditions: denaturation 95°C 10 min; amplification 95°C 10 sec, 60°C 10 sec, extension 72°C 10 sec, cycle 45; final extension 72°C 10 min. Quantification was performed using a standard curve and analyzed by the ABI StepOnePlus software. For qPCR analysis the following sequence of forward (F) and reverse (R) primers were used. IL-8.F:5′-CTGCGCCAACACAGAAATTATTGTA-3′; R:5′-TTCACTGGCATCTTCACTGATTCTT-3′ PTHrP.F:5′-AGAGCAGCCGCTCAAGACAC-3′, R:5′-GGTGGTCCCCTTCTAGCCCA-3′; TGFβ.F:5′-CAAGGACCTCGGCTGGAA-3′, R:5′-CCGGGTTATGCTGGTTGTACA-3′ RANKL.F:5′-ACCAGCATCAAAATCCAAG-3′, R:5′-CCCCAAAGTATGTTGCATCC-3′ MMP9.F:5′-ATTTCTGCCAGGACCGCTTCTACT-3′, R:5′CAGTTTGTATCCGGCAAACTGGCT-3′.

### Immunofluorescence

PC-3 cells and cells transfected with vector alone or DN-LRP5 plasmid were grown overnight in 24-well plates. Cells were fixed in 3% paraformaldehyde for 15 min at room temperature followed by two washes in ice cold phosphate-buffered saline (PBS). Cells were permeabilized in 0.1% Triton X-100 for 10 min at room temperature followed by three washes in PBS. Cells were blocked with 1% bovine serum albumin (BSA) in PBS for 30 min followed by an overnight incubation at 4°C with β-catenin antibody (ABM, Richmond, BC). After three washes in PBS, the cells were incubated with Cy3 conjugated goat anti-rabbit IgG (1:400) for 1 h in the dark. Nuclei were stained with SYBR green (1:20,000). The cells were then washed with PBS and mounted with Mowiol. The fluorescence-stained cells were examined with a Zeiss LSM 510 confocal microscope (Carl Zeiss, Toronto).

### Cell invasion, colony formation, and wounding assay

The invasive capacity of 5 × 10^4^ PC-3-pcDNA3.1 and PC-3-DN-LRP5 cells were examined using two-compartment Boyden chamber Matrigel invasion assay (Costar Transwell, Corning Corporation, Corning, NY) as described previously 37. For colony formation assay of PC-3-pcDNA3.1 and PC-3-DN-LRP5, 3 × 10^3^ cells, were seeded in triplicate onto six-well petri dishes in the presence of 4 mL of culture medium containing 1.5% agar solution at 37°C. Medium was changed every 48 h, and the number of colonies were scored as >100 cells after 14 days of plating 38.

For colony formation assay of PC-3-pcDNA3.1 and PC-3-DN-LRP5, 3 × 10^3^ cells were seeded in triplicate onto six-well petri dishes in the presence of 4 mL of culture medium containing 1.5% agar solution at 37°C. Medium was changed every 48 h, and the number of colonies were scored as >100 cells after 14 days of plating 38.

Both PC-3-pcDNA3.1 and PC-3-DN-LRP5 cells grown in the presence of 10% fetal bovine serum (FBS) and then plated in six-well plates to form a monolayer. The wound was done manually with a sterile 1000 μL pipette tip in the center of each well 37. Cells were grown in the presence of 2% FBS and at different time points the migrating cells were photographed, selected for analysis, and quantified using Image Pro-Plus software and calculated as percentage wound healing using the equation% wound healing = (1−[wound area at *T*x h/wound area at *T*_0_]), wherein *T*x is the respective time point and *T*_0_ is the time immediately after wounding. These experiments were repeated twice in duplicates.

### Animal protocols

Six-week-old male Balb C nu/nu mice were obtained from NCI Research Resources, Frederick, MD. Before inoculation, PC-3-pcDNA3.1 and PC-3-DN-LRP5 cells growing in serum-containing medium were washed with Hank's balanced buffer, trypsinized, and centrifuged at 1500 rpm for 5 min. Cell pellets were resuspended in 200 μL of saline with 20% Matrigel. An anesthetic cocktail of ketamine (50 mg/kg), xylazine (5 mg/kg), and acepromazine (1 mg/kg) was injected intramuscularly, and 2 × 10^6^ cells were inoculated using a 26-gauge needle subcutaneously (s.c.) into the right flank of anesthetized mice. Both control and experimental animals were monitored at weekly intervals for 7 weeks for tumor development and growth. Tumor volume (TV) was determined according to the formula: TV = (shorter diameter)^2^ × longer diameter/2. Results were presented as the mean of TVs recorded from all animals within a particular cohort. At the end of the study, animals were sacrificed, and primary tumors were removed. Half of the tumors were fixed in 10% buffered formalin for immunohistochemical analysis, and the other half were snap frozen in liquid nitrogen.

For *in vivo* studies, PC-3-pcDNA3.1 cells and PC-3-DN-LRP5 were grown in RPMI+10% FBS. At confluence, cells were harvested and the cell pellets were washed with sterile saline and centrifuged at 1500 rpm for 5 min. Six-week-old male Fox Chase severe combined immune deficient (SCID) mice were obtained from Charles River. Following the administration of an anesthetic cocktail of ketamine (50 mg/kg), xylazine (5 mg/kg), and acepromazine (1 mg/kg) intramuscularly, PC-3-pcDNA3.1 and PC-3-DN-LRP5 cells were inoculated at 2 × 10^5^ cells per mouse in 40 μL saline with a 27-gauge needle into the left tibia using a drilling motion. The mice were monitored weekly for tumor burden. On week 4, a digital radiography of hind limbs of all animals was done using a Kubtec digital X-ray 37 to monitor the development of skeletal lesions. On week 4, the mice were euthanized, and the left tibias were collected and fixed in 10% buffered formalin solution for 24 h. The X-ray scoring method is described as follows: no lesions, minor changes, small lesions, significant lesions (minor peripheral margin breaks, 1–10% of bone surface disrupted), and significant lesions (major peripheral margin breaks, >10%) of bone surface broken rating 0–4, respectively 37. The whole tibia (trabecular and cortical) of four different animals from each group were analyzed by microcomputed tomography with a SkyScan 1072 scanner and associated analysis software (SkyScan, Kontich, Belgium). Image acquisition was done at 45 kV with a 0.9 rotation between frames. During scanning, the tibias were enclosed in a tightly fitted plastic wrap to prevent movement and dehydration. All the experimental animal protocols were in accordance with the McGill University Animal Care Committee guidelines.

### Statistical analysis

Results were analyzed as the mean ± SE, and comparisons of the experimental data were analyzed by an independent two-sample *t-*test at *P *< 0.05 level of significance.

## Results

### Effect of DN-LRP5 on epithelial, mesenchymal markers, and β-catenin expression in PC-3 cells

**Figure 1:**
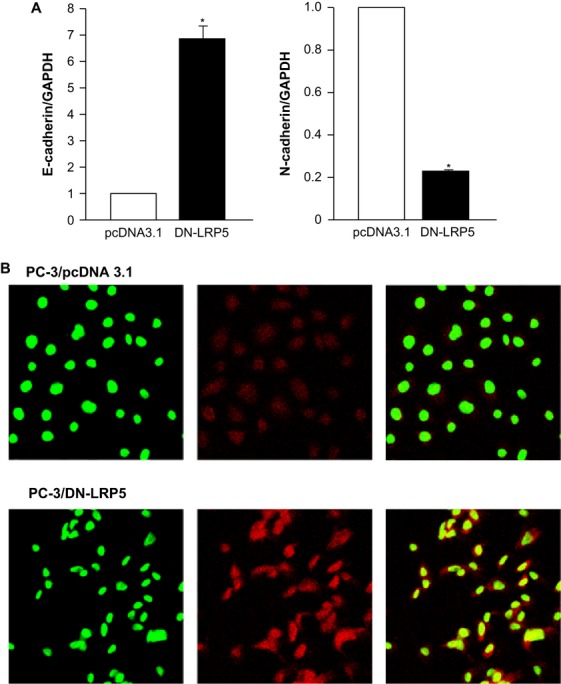
Effect of DN-LRP5 on mesenchymal to epithelial shift and cytoplasmic translocation of β-catenin in human prostate cancer cells PC-3. (A) Control (PC-3-cDNA3.1) and experimental (PC-3-DN-LRP5) PC-3 cells were grown to confluence and total cellular RNA was isolated with TRIzol. Changes in the mRNA expression of the N and E cadherin was determined by real-time PCR by plotting the relative ratio against glyceraldehyde 3-phosphate dehydrogenase (GAPDH) using primers described in “Material and Methods”. Results are representative of at least two different experiments, where white bars represent controls and solid black bars represent experimental cells. Significant difference from the control is represented by an asterisk (*P < 0.05). (B) Control (PC-3-pcDNA3.1) and experimental (PC-3-DN-LRP5) cells were subjected to immunostaining for the change in levels and site of β-catenin expression. In control cells expression of β-catenin was predominantly seen in the PC-3 cell nuclei. In contrast in experimental PC-3-DN-LRP5 cells, β-catenin expression was mostly located in the cellular cytoplasm.

Human PCa cells PC-3 were transfected with vector alone and DN-LRP5 plasmid were maintained in culture. Total cellular RNA was extracted and change in the levels of expression of epithelial (E-cadherin) and mesenchymal marker (N-cadherin) was determined by qPCR as described in “Material and Methods”. PC-3-DN-LRP5 cells showed a significant (sixfold) increase in E-cadherin and decrease (78%) in N-cadherin mRNA expression. Results from these studies confirmed the transition of experimental PC-3 cells from an epithelial to mesenchymal phenotype (Fig. 1A). Control (PC-3-pcDNA3.1) and experimental (PC-3-DN-LRP5) cells were subjected to immunostaining for the change in levels and site of β-catenin expression. In control cells expression of β-catenin was predominantly seen in the PC-3 cell nuclei. In contrast in experimental PC-3-DN-LRP5 cells, β-catenin expression was mostly located in the cellular cytoplasm (Fig. 1B). The translocation of β-catenin to the cytoplasm resulted in blocking the effects of β-catenin via the conical signaling pathway in experimental PC-3 cells.

### Effect of DN-LRP5 on PCa cells invasion and colony formation

**Figure 2:**
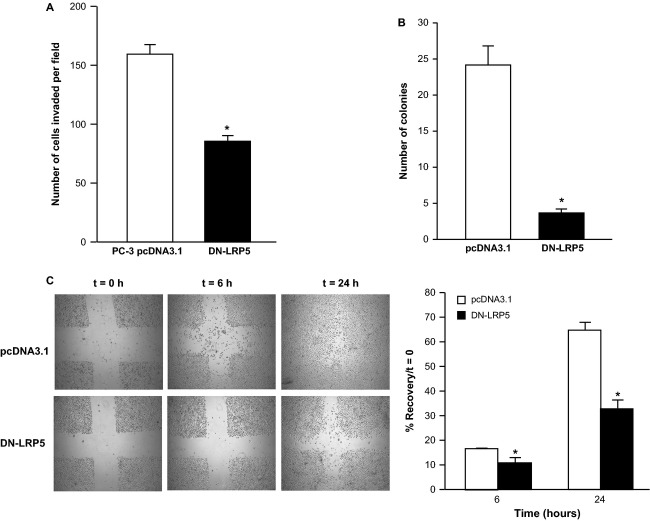
Effect of DN-LRP5 on PC-3 cells invasion, colony formation, and migration *in vitro*. Control (PC-3-pcDNA3.1) and experimental (PC-3-DN-LRP5) cells invasive capacity was evaluated by using a Boyden chamber Matrigel invasion assay. After 18 h of incubation, the invaded cells were fixed, stained, and 10 random fields were counted. Number of cells invading is shown as bar diagram ± SEM (A) as described in “Material and Methods”. Using soft agar assay, the ability of control and experimental cells to form colonies was examined and the number of colonies formed in each group was counted (B) as described in “Material and Methods”. PC-3 cells migration was determined by wound healing assay by seeding PC-3-pcDNA3.1 and PC-3-DN-LRPP5 cells in six-well plates and allowing them to grow as a monolayer and making a wound as described in “Material and Methods”. Control and experimental migrating cells were photographed at different time points (C). Percent wound healing was recorded at different time points, and percentage of wound healing with respect to *T*_0_ was calculated using the equation described in “Material and Methods”. Results are presented as the mean ± SEM of two different experiments in duplicate from control and experimental cells. Significant differences from the control is represented by an asterisk (*P *< 0.05).

The effect of transfection of DN-LRP5 on PC-3 cells invasive capacity was examined by Matrigel invasion assay. Control PC-3 showed high invasive capacity. However, cells transfected with DN-LRP5 plasmid showed a significant inhibition (46%) in their invasive capacity (Fig. 2A). In these studies we also determined the number of tumor cells in both upper and lower part of Boyden chamber which showed similar number of control and experimental tumor cells during these studies.

We next examined the effect of DN-LRP5 transfection on the ability of experimental PC-3 cells to form colonies in soft agar. Control vector transfected cells formed 25 colonies per field which was similar to that seen in wild-type cells (data not shown). However, experimental PC-3-DN-LRP5 cells showed as marked decreased (85%) in the number of colonies formed per field of examination (Fig. 2B).

### Effect of DN-LRP5 on PCa cell migration

The effect DN-LRP5 plasmid transfection on cell migration was examined as described in “Material and Methods”. Control PC-3 cells showed the ability of these cells to start migration at 6 h with significant closure of margins at 24 h. In contrast the experimental PC-3-DN-LRP5 cells continued to show a significant decrease in their ability to migrate as shown by representative micrographs which were also quantified (Fig. 2C). Results from these studies demonstrate that by inhibition of β-catenin pathway by transfection of DN-LRP5 plasmid results in a marked increase in the ability of tumor cells to proliferate, but a decrease in the ability to invade and migrate *in vitro*.

### Effect of DN-LRP5 on PC-3 on tumor growth and experimental skeletal metastasis *in vivo*

**Figure 3:**
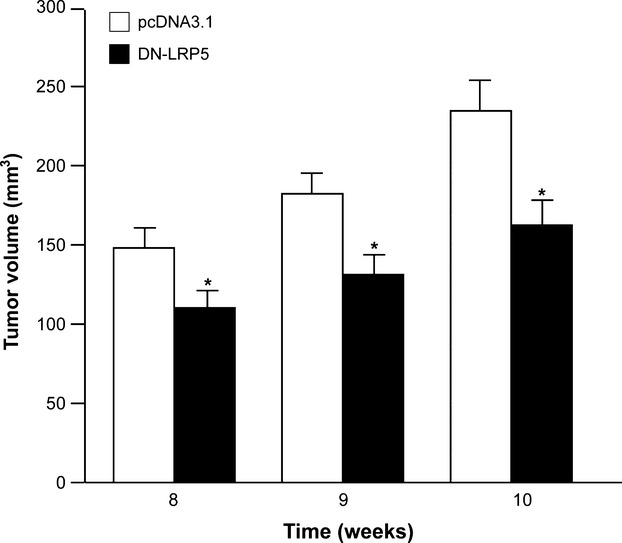
Effect of DN-LRP5 on prostate cancer growth *in vivo*. Male Balb C nu/nu mice were inoculated with (2 × 10^6^) control (PC-3-pcDNA3.1) and experimental (PC-3-DN-LRP5) PC-3 cells through the s.c. route. Tumors were measured weekly, and tumor volume was determined as described in “Material and Methods”. Result represents the mean ± SEM of eight animals in each group. Significant differences from control are represented by asterisks (*P < 0.05).

The effect of transfection of DN-LRP5 plasmid into PC-3 cells was examined by injecting control and experimental PC-3 cells into the right flank of male Balb c nu/nu mice. Tumors were measured at weekly intervals and comparison was made between control and experimental animals. Transfection of DN-LRP5 plasmid resulted in the development of tumors of significantly smaller volume throughout the course of these studies as compared with control group of animals inoculated with PC-3 cells transfected with vector alone (Fig. 3).

**Figure 4:**
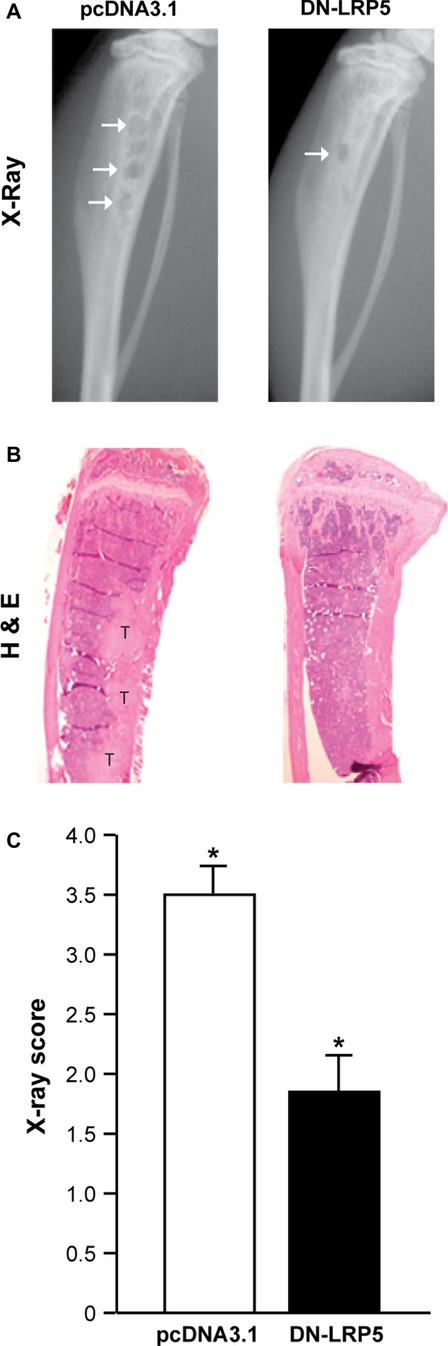
Effect of DN-LRP5 on prostate cancer skeletal lesions *in vivo*. Male Fox Chase SCID mice were inoculated with (2 × 10^5^) control (PC-3-pcDNA3.1) and experimental (PC-3-DN-LRP5) PC-3 cells via i.t route. Development of skeletal lesions was determined at weekly intervals by X-ray using Kubtec digital X-ray, and lesion area was determined as described in “Material and Methods”. Skeletal lesions in radiographs are highlighted by arrows, and histologic analysis was carried by hematoxylin and eosin (H&E) staining where i.t. tumors are marked as “T”. Representative radiograph lesion score of control and experimental animals at week 4 following tumor cell inoculation is shown in lower panels. Result represents the mean ± SEM of eight animals in each group. Significant differences from control are represented by asterisks (*P *< 0.05).

**Figure 5:**
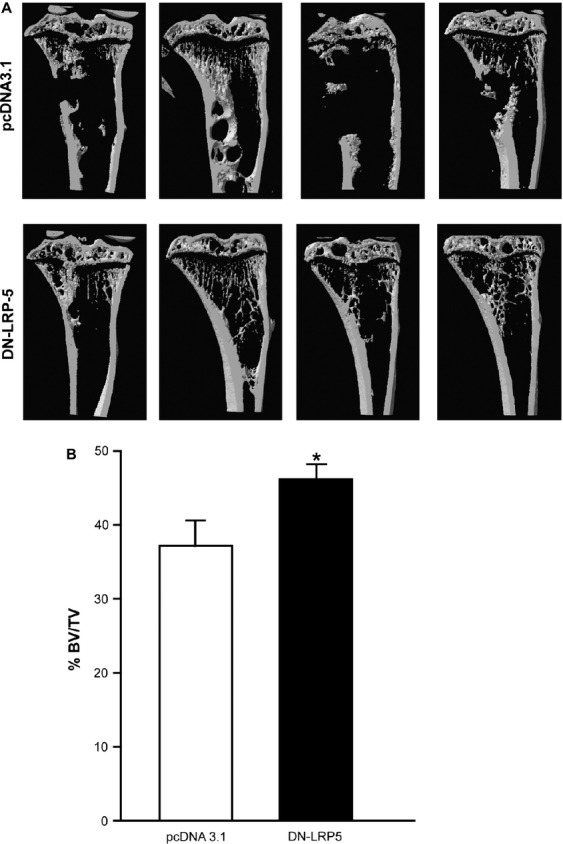
Effect of DN-LRP5 on prostate cancer skeletal lesion *in vivo* by microcomputed tomography (μCT). Male Fox Chase SCID mice were inoculated with (2 × 10^5^) control (PC-3-pcDNA3.1) and experimental (PC-3-DN-LRP5) PC-3 cells via i.t route. At week 4 post tumor cell inoculation, all animals were sacrificed and their tibias were collected and fixed. Tibias from four representative animals in each group underwent analysis by μCT with SkyScan and the frontal view is shown (A). The % bone volume to tumor volume ratio (BV/TV), trabecular spacing, and connectivity density (B) were determined by the Skyscan software as described in “Material and Methods”. Significant differences from control are represented by asterisks (*P < 0.05).

A number of genes which are implicated in tumor growth and bone biology are regulated by the β-catenin signaling pathway which can affect the ability of tumor cells to form skeletal metastases in our xenograft model. In order to monitor the effect of blocking this pathway by transfection of DN-LRP5 plasmid control and experimental PC-3 cells were injected into the tibia of male Fox Chase SCID mice. Radiological examination of all animals was carried out at timed intervals by Kubtec digital X-ray (Fig. 4A). At the end of these studies all animals were sacrificed and their tibia removed. Histological analysis of these control and experimental tibia following hematoxylin and eosin (H&E) staining confirmed this significant decrease in skeletal lesions of experimental animals (Fig. 4B). These studies showed a marked decrease in the number and area of skeletal lesions in experimental animals inoculated with PC-3-DN-LRP5 cells as compared to vector transfected PC-3-pcDNA3.1 cells (Fig. 4C). At least four tibias of representative control and experimental groups were subjected to μCT analysis. Results from these studies showed significant increase in bone volume (BV) to TV ratio in tibias from experimental animals to show that net decrease in skeletal lesions resulted in increased BV (Fig. 5).

### Effect of DN-LRP5 on PCa-associated gene expression

**Figure 6:**
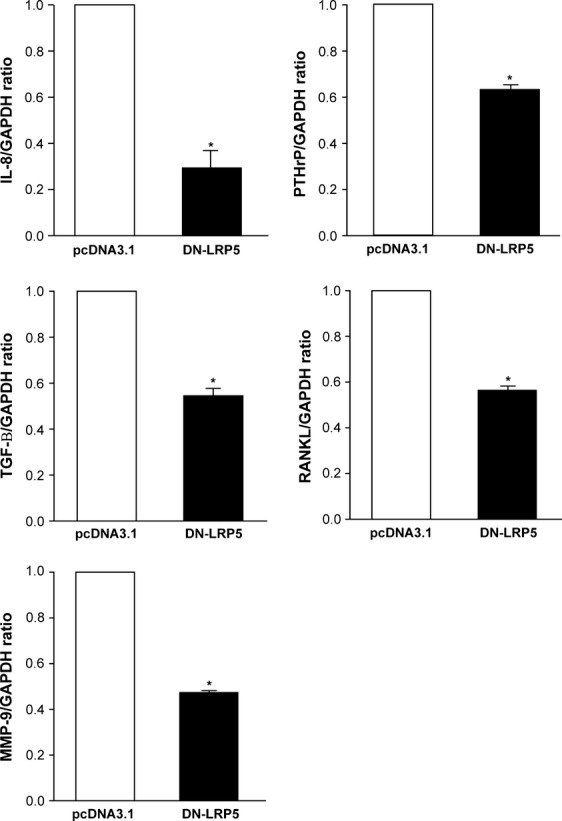
Effect of DN-LRP5 on the expression of genes associated with prostate cancer progression and skeletal metastasis. Control (PC-3-pcDNA3.1) and experimental (PC-3-DN-LRP5) PC-3 cells were grown to confluence and total cellular RNA was isolated with TRIzol. Changes in the mRNA expression of the representative genes implicated in prostate cancer-associated skeletal metastasis were determined by real-time PCR by plotting the relative ratio against GAPDH using primers described in “Material and Methods”. Results are representative of at least two different experiments, where white bars represent controls and solid black bars represent experiment cells. Significant difference from the control is represented by an asterisk (*P < 0.05).

The molecular mechanism of these *in vitro* and *in vivo* effects seen in experimental cells was examined by evaluation of the levels of expression of key genes involved in tumor progression and bone biology. Total cellular RNA was extracted from PC-3-pcDNA3.1 and PC-3-DN-LRP5 cells and subjected to real-time PCR (qPCR) using the primers listed in “Material and Methods”. Results from these studies shown in Figure 6 show a significant decrease in the levels of mRNA expression of interleukin 8 (IL-8) parathyroid hormone-related peptide (PTHrP), transforming growth factor β (TGF-β), receptor activator of nuclear factor kappa-B ligand (RANKL), and matrix metalloprotease 9 (MMP-9). Among these genes IL-8, PTHrP, TGF-β, and RANKL are extensively described for their role in bone biology whereas PTHrP, TGF-β, and MMP-9 are characterized for their involvement in tumor progression and skeletal metastasis.

## Discussion

In the current studies, we extended our previous finding to demonstrate the role of β-catenin signaling pathway in PCa 34. As skeletal metastasis is a major complication associated with late-stage hormone refractory PCa patients, our focus was to investigate the effect of knockdown of this pathway in the development and progression of skeletal metastasis. Toward these goals we used the well-characterized dominant negative knockdown of LRP5 which has a profound effect on the role of LRP5 in effective signaling of the β-catenin pathway. Among the various human PCa cell lines examined, we have previously shown the highest level of β-catenin in human PCa cells PC-3 cells which best mimic the late stages of human PCa 34. Following the transfection of DN-LRP5 plasmid a significant change in PC-3 cell characteristics was seen which reflected PC-3 cells reversal from epithelial to mesenchymal phenotype. This change was also associated with a shift in the location of β-catenin from the PC-3 cell nucleus to the cytoplasm. These well characterized PC-3-DN-LRP5 cells showed a significant decrease in cell invasion migration and colony formation in our well characterized *in vitro* assays. These characteristics are directly related to the ability of tumor cells to grow and acquire metastatic potential. Using our well established xenograft model of PCa, LRP5 knockdown resulted in the development of tumors of a significantly smaller volume, effects which were efficiently maintained for 10 weeks post tumor cell inoculation. We then examined the ability of experimental PC-3-DN-LRP5 cells to form skeletal lesions in our experimental model of skeletal metastasis. Using multiple approaches including X-ray, histology, and μCT, animals inoculated with PC-3-DN-LRP5 cells consistently showed a significantly lower number and area of skeletal lesions. While these changes were significant and consistent, using this approach did not completely knock down this key signaling pathway implicated in tumor progression 3–7. This is in line with the use of additional and or alternate molecules which can continue to activate β-catenin signaling 9–11. Nevertheless taken together results obtained in these studies showed a significant role of LRP5 in modulating primary tumor growth and in the development of skeletal lesions. In order to elucidate the molecular mechanism of these effects, we examined the change in the levels of expression of genes which are well described for their role in the development and progression of skeletal metastasis 3–7. The change in BV observed following the inoculation of PC-3-DN-LRP5 cells is also consistent with several reports where β-catenin altered osteoblast proliferation and bone formation 35–39. Blocking the wnt pathway can affect a large number of genes. However, we have focused on the change of expression of key genes previously implicated in PCa -associated skeletal metastases. Results obtained by qPCR showed a marked decrease in the expression of IL-8, PTHrP, TGF-β, RANKL, and MMP-9, all of which are involved in tumor cell invasion and skeletal metastasis.

Bone remodeling is a key component which regulates the degree and severity of skeletal related events like pain, nerve compression associated with skeletal metastasis 4,5. Targeting of signaling pathways like wnt-β-catenin where members of the wnt family are both oncogenic and osteo-inductive supports their key role in skeletal metastasis 40,41. While a number of studies have demonstrated the multiple roles of this pathway in tumor progression where transfection of β-catenin siRNA into colon and breast cancer cells led to the induction of metastatic genes whereas stabilization of β-catenin by lithium chloride or treatment with glycogen synthase kinase-3b (GSK-3b) inhibitor decreased their expression 40–42. These studies led the authors to propose the cautionary use of β-catenin inhibitors as anticancer therapy for a broader patient population 42. In summary downregulation of these pro-metastatic genes by β-catenin knockdown provides further support for the ability of Wnt/β-catenin signaling to regulate the multistep process of tumor progression and rationale for targeting this pathway in malignancy. With the identification of the role of wnt pathway in tumorigenesis several inhibitors targeting this pathway are being developed. However, the potential toxicity due to nonspecific inhibition must be considered as these reagents are developed for their clinical application 43. While the impact of these findings in patients with PCa remains to be determined, combined with our previous studies and data from other laboratories, there is now accumulated and compelling evidence for targeting LRP5-mediated wnt/β-catenin pathway as a diagnostic, prognostic, and therapeutic target in patients with PCa and other common malignancies associated with skeletal metastases.

## Conflict of Interest

None declared.
